# The lifestyle habits and wellbeing of physicians in Bahrain: a cross-sectional study

**DOI:** 10.1186/s12889-015-1969-x

**Published:** 2015-07-14

**Authors:** Saif M Borgan, Ghufran A Jassim, Zaid A Marhoon, Mahmoud H Ibrahim

**Affiliations:** Arab Medical Center, Amman, Jordan; Department of Family and Community Medicine, Royal College of Surgeons in Ireland- Medical University of Bahrain, P.O. Box 15503, Adliya, Kingdom of Bahrain; Salmaniya Medical Complex, Manama, Bahrain; King Hamad University Hospital, Manama, Bahrain

**Keywords:** Lifestyle, Wellbeing, Health, Physicians, Practitioners, Exercise, Physical activity, Body mass index, Alcohol consumption, Diet

## Abstract

**Background:**

Lifestyle habits of physicians are of paramount importance both because they influence the physician’s own health and because these habits have been shown to affect patients’ care. There is limited information on physician health and lifestyle habits in Bahrain.

**Methods:**

In a cross-sectional study design, an anonymous self-administered questionnaire that assesses wellbeing and lifestyle habits was distributed to a random sample of 175 out of 320 primary health care physicians in Bahrain. Descriptive analyses were performed, and the variables were cross-tabulated using SPSS version 20.0.

**Results:**

152 physicians agreed to participate in the study. Respondents were 67.1 % female with a mean age of 45 (SD = 10). The majority were of Bahraini nationality. The most prevalent reported health conditions were hyperlipidaemia (25.5 %), hypertension (20.3 %), and diabetes (11.0 %). Only 29.6 % of physicians reported performing ≥ 30 min of exercise in a usual week. Of physicians exercising ≥ 30 min weekly, only 13 % exercised ≥ 5 days weekly. 98.0 % report never drinking, 1.3 % report previously drinking, and 0.7 % report drinking less than once weekly. The average body mass index (BMI) was 27.8 (SD = 5), with 39 % of physicians being overweight and 33 % obese. BMI was directly associated with sleep time (P0.027, r^2^ = 0.034), age (P < 0.01, r^2^ = 0.179), male gender (P = 0.031, r^2^ = 0.054), and a known diagnosis of hypertension (P = 0.007, r^2^ = 0.079) or hyperlipidaemia (P = 0.008, r^2^ = 0.088).

**Conclusions:**

There is a clear pattern of unfavourable lifestyle habits and obesity among primary health care physicians in Bahrain. We encourage institutions and public health sectors to be more proactive in assisting physicians to attain healthier lifestyles.

**Electronic supplementary material:**

The online version of this article (doi:10.1186/s12889-015-1969-x) contains supplementary material, which is available to authorized users.

## Background

The World Health Organization defines health as a state of complete physical, mental and social wellbeing, and not merely the absence of disease or infirmity [1]. Healthy lifestyle habits including regular physical activity, a balanced diet, and refraining from smoking and excessive alcohol consumption have been shown to reduce the overall mortality from non-communicable disease (NCD), which accounts for 36 million deaths worldwide yearly [2]. The 2010 NCD report published by the Ministry of Health in Bahrain presents alarming data regarding the lifestyle habits of the Bahraini population [3]. The average body mass index (BMI) in Bahrain was found to be 28.5, with levels of obesity and overweight reaching 36.3 % and 32.9 %, respectively. Other lifestyle habits appear to be just as unfavourable. Only 43.0 % of the population (age group 20–65) reported exercising during their free time. Smoking was more prevalent among males than females, with 33.4 % of male adults smoking tobacco regularly [3]. The impact of these lifestyle habits is evident in the measured rates of hypertension (42.7 %), hypercholesterolemia (40.2 %) and diabetes mellitus (14.5 %) among the general population [3]. Another concerning measure is the Bahraini mean fasting blood sugar level (mFBS), which according to the WHO, has increased from 5.4 mmol/1 (males) and 5.1 mmol/l (females) in 1980 to 5.9 mmol/l (males) and 5.7 mmol/l (females) in 2009 [4]. This gradual rise in blood sugar predisposes to higher rates of future diabetes mellitus and its associated medical complications in the general population.

Physicians have the privilege and responsibility of taking a frontline role in promoting healthy lifestyle habits, because they have daily opportunities to consult with their patients. One study in Bahrain has linked physicians’ advice on smoking cessation with a positive attitude toward quitting smoking [5], while studies in other countries have confirmed the effectiveness of physicians’ advice on lifestyle habits for inducing and maintaining positive behaviour changes in patients [6–8]. One of the strongest predictors of physicians giving lifestyle advice is the physician’s own health behaviour [9].

Physicians’ lifestyle habits are of particular importance both because they influence the physician’s own health and because that these habits affect physician–patient consultations. Physicians with healthy practices are more likely to discuss related preventative measures with their patients, while physicians with unfavourable lifestyle habits are less proactive in giving advice that they do not follow themselves [10, 11]. This phenomenon could be explained by the association between the health behaviours that physicians themselves struggle to attain and the effects of these behaviours on both, physicians’ confidence to counsel their patients and their actual counselling practices [11].

Previous studies in Bahrain have addressed some physician habits. Two studies assessed the prevalence of tobacco smoking, revealing smoking rates of 26.6 % among male physicians and 24 % overall in 1991 and 2009, respectively [12, 13]. High rates of tobacco smoking among physicians in Bahrain were partly attributed to the rising prevalence of waterpipe smoking and the lack of education and awareness regarding the health effects of tobacco smoking [13, 14]. A 2003 study showed that only 29.7 % of physicians exercise during their leisure time and that their mean BMI is 26.8 kg/m2 [15]. However, no studies have evaluated associations between lifestyle habits and physician wellbeing in Bahrain, and many of these studies are out of date because the number of physicians in Bahrain has been rapidly increasing to accommodate the expatriate population boom. We conducted a cross sectional study to explore physician general wellbeing and lifestyle habits including exercise, alcohol consumption, reported sleep time, smoking habits, perceived subjective stress levels (on a scale from 0 to 10), and screen time (time spent using computers and TV’s).

## Methods

### Population and sampling

We first established the total population of primary health care physicians in Bahrain. A list of all full time primary care physicians was acquired from the Ministry of Health (N = 320). A computerised random sample generator selected a sample of 175 physicians distributed throughout all 27 primary healthcare service locations nationwide. This sample size was estimated to give a 90 % confidence interval of within 5 % of the true value, allowing for a 15 % non-response rate. From July to August 2013, physicians were personally approached during their allocated break time and were asked to take part in the study.

### Questionnaire

A previously developed self-administered questionnaire (Additional file 1) [16] was used to assess the wellbeing and lifestyle habits of primary care physicians in Bahrain. The survey questionnaire, which was distributed in paper form, has three main sections assessing physician demographics, wellbeing, and lifestyle habits. The wellbeing section assessed BMI, perceived health status, health care visits, dentist visits, hospital admissions, current health conditions, physical disabilities and psychiatric disorders. BMI was calculated by asking physicians about their height in cm and weight in kg. Perceived health status was assessed by asking “Your health in general is?” with categories for excellent, good, fair and poor. Health care visits were assessed by asking “In the previous 6 months, have you seen any health professional regarding your own health?” and those who answered yes were asked to specify the reason for visit. Current self reported health conditions were assessed by asking “Have you ever been told by a doctor that you have one of the following health conditions?” followed by a table of the common health conditions with a yes or no option. Physicians were also asked to specify “Other health conditions” which were not included in the table. Other questions in the wellbeing section included close ended questions on physical disability“ Do you have any physical disability?” and psychiatric conditions “Do you have any psychiatric conditions?” . Lifestyle habits section included physical activity, nutrition, alcohol consumption, illicit drug use, sleep, stress, screen time and driving behaviours. Physical activity was assessed through 4 questions; “ During a usual week, do you practice any sports, exercise or other physical activity for at least 30 min in duration and in sufficient intensity to induce perspiration?” and two follow up questions, one close ended “ During a usual week, how many days do you practice these activities?” and one open ended “What type of sport, exercise or other physical activity did you practice in the last week?”. The last question on activity level asked “Do you usually use the elevator or stairs in multi-story buildings?” with categories for elevator, stairs and both. Sleep was assessed through an open ended question “Usually, how many hours do you sleep per night?” Stress was assessed by asking participants to rate how stressed they feel in their lives on a scale with increasing intensity from 0 to 10. Screen time was assessed by asking the following question “ What is your average daily time duration of using the following?” to both TV/DVD and Laptop/computer with categories for 1–2 or less hours, 3–5 h and 6–10 or more.

### Statistical analysis

Descriptive analysis was performed on all variables. Fisher’s exact tests were used to test associations between categorical variables, while Pearson correlations were used to test associations between continuous and categorical variables. SPSS version 20.0 was used in all analyses with significance set as p < 0.05.

### Ethical consideration

Ethics approval was obtained from RCSI–Bahrain Research Ethics Committee and the Ministry of Health in Bahrain. Written consent was obtained from all physicians prior to participation in the study.

## Results

### Population characteristics

One hundred and fifty two physicians agreed to participate in the study (86.9 % response rate). Table 1 shows sample demographics including age, gender, nationality, and marital status. Participants had a mean age of 45 years (SD = 10) and 67.1 were female. The majority of physicians (77.3 %) specialised in family medicine.

**Table 1:** Population characteristics

Characteristic	No.	%
***Age***		
30-39	53	37
40-49	42	29
50-59	35	24
>60	15	10
***Gender***		
Male	50	33
Female	102	67
***Nationality***		
Bahraini	117	77
Non-Bahraini	34	23
***Marital status***		
Married	142	95
Single	8	5
***Specialization***		
Family medicine	116	77
General practice	34	23

### Wellbeing

Twenty six per cent of physicians consider their health status “excellent”, 66 % “good”, 7 % “fair”, and 1 % as “poor”. The prevalence of known medical conditions among physicians is as follows: hyperlipidaemia (25.5 %), hypertension (20.3 %), hereditary blood disorders (15.8 %), diabetes (11.0 %), asthma (9.7 %), heart conditions (3.4 %), and psychiatric conditions (1.3 %). Individuals with a known diagnosis of diabetes, hyperlipidaemia, or hypertension are more likely to have a negative perception of their health status (P = 0.02, 0.012 and <0.01 respectively). Seven per cent of individuals reported having a physical disability. Only 49.3 % of physicians reported visiting a dentist and 36.9 % reported visiting a medical doctor in the past six months. The majority of doctor visits in the previous 6 months were to obstetrics and gynaecology or surgical specialists (61.9 %), while internal medicine and its subspecialties constituted 38.1 % of all visits (14 % of all physicians). Only 3/152 physicians specifically reported visiting a physician in the previous 6 months to screen or check on a chronic disease. The average physician BMI was 27.8 [SD = 4.6]. Two physicians (1.4 %) were underweight, 26.1 % had a normal BMI, 39.4 % were overweight, and 33.1 % were obese. Physicians with an above normal BMI (>25) were more likely to be males (P = 0.031), older in age (P < 0.01), report less than six sleep hours nightly (P0.027), and have a known diagnosis of hypertension (P = 0.007) or hyperlipidaemia (P = 0.008). However, only age was a significant independent predictor of BMI (P <0.01, r^2^ = 0.179). Table 2

**Table 2:** Cross tabulation of BMI with self reported medical conditions and selected lifestyle habits

		BMI category				
		Average or below		Above average		Statistics^b^
		n	(%)	n	(%)	
Age Groups	30-39	22	(46.8)	25	(53.2)	*p* < .001, r^2^ = 0.179
	40-49	13	(32.5)	27	(67.5)	
	50-59	1	(2.9)	33	(97.1)	
	60-69	0	(.0)	15	(100.0)	
Gender	Male	8	(16.3)	41	(83.7)	*p* = 0.31, r^2^ = 0.054
	Female	31	(33.3)	62	(66.7)	
Heart Disease^a^	Yes	0	(.0)	5	(100.0)	*p* = .156
	No	38	(29.0)	93	(71.0)	
Diabetes Mellitus^a^	Yes	1	(6.7)	14	(93.3)	*p* = .052
	No	37	(30.6)	84	(69.4)	
Hyperlipidaemia^a^	Yes	4	(10.8)	33	(89.2)	*p* = .008, r^2^ = 0.088
	No	34	(33.3)	68	(66.7)	
Hypertension^a^	Yes	2	(7.1)	26	(92.9)	*p* = .007, r^2^ = 0.079
	No	36	(32.7)	74	(67.3)	
Hereditary Blood Disease^a^	Yes	9	(42.9)	12	(57.1)	*p* = .098
	No	29	(25.2)	86	(74.8)	
Exercise	Yes	8	(19.5)	33	(80.5)	*p* = .176
	No	31	(30.7)	70	(69.3)	
smoker	Yes	3	(25.0)	9	(75.0)	*p* = .830
	No	36	(27.9)	93	(72.1)	
Sleep hours	<6	7	(15.9)	37	(84.1)	*p* = .027, r^2^ = 0.034
	≥6	32	(34.0)	62	(66.0)	
Fast Food Portion per week	None	24	(31.2)	53	(68.8)	*p* = .282
	At least once	15	(23.1)	50	(76.9)	

^a^Self-Reported

^b^Pearson Chi Square for P Values

Linear Regression Analysis for r^2^

### Exercise levels

Only 29.6 % of physicians reported performing ≥30 min of continuous physical activity during an entire week. Of those who exercise weekly, the majority (81 %) exercise 3 days per week or less. The most commonly reported exercises were walking and swimming. Physicians who were physically active during the week were significantly more likely to report lower stress levels (P = 0.029) and significantly more likely to refrain from consuming fast food during the week (P = 0.035). Few physicians (27.8 %) reported always using the stairs in multi-story buildings; 27.2 % reported always using the elevator, while 45.0 % reported using the stairs at certain times.

### Smoking habits, alcohol consumption and drug use

Results from the smoking section of this study have been published separately [17]. They show that the prevalence of waterpipe smoking exceeds cigarette smoking and that male physicians are the predominant smokers [17]. The majority of physicians (98.0 %) reported never drinking alcohol, while former drinkers comprised 1.3 % of the population, and current drinkers comprised only 0.7 %. Only one physician (0.7 %) reported illicit drug use. Two physicians (1.3 %) reported having used stimulant pharmacological agents such as amphetamines in the preceding 6 months.

### Diet and nutrition

Forty one per cent of the population eat all three main meals every day. Breakfast is the most frequently neglected meal during a typical week. Information on fruit, vegetable and meat consumption is provided in **the supplementary material section**. Eighty one physicians (53.3 %) reported not eating fast food meals during the week, while 28.9 % eat fast food once per week, 15.8 % twice per week, and 2 % most days of the week. The mean daily water intake was 1.7 l (SD = 1.1). Forty four per cent of physicians consume at least one carbonated soft drink daily. A majority of the sample (86.2 %) consume at least one caffeinated beverage daily, excluding soft drinks. Forty seven per cent of physicians have been on a diet to reduce their weight in the last 6 months, and 6.6 % have taken medication to help them lose weight.

### Sleep and stress

The mean reported daily sleeping time was 5.9 h (SD = 1.2). The majority of physicians (86.3 %) report less than 8 h of sleep, and 30.8 % report less than 6 h of sleep. Physicians who smoke were significantly more likely to report a total sleeping time of less than six hours daily (P = 0.004). Those who reported 6 h or more of sleep time were more likely to consume breakfast on most days of the week (P = 0.035). The mean physician rating of perceived stress level was 6.4/10 (range = 1–10, median = 7, SD = 2.0). Perceived stress levels [1–5] were considered low, [6–8] moderate, and [9, 10] severe. Females were significantly more likely to report severe stress levels than males (P = 0.001).

### Screen time

Reported computer screen time was higher than television screen time, with 62.5 % using computers for 2 h or less, 22.2 % for three to five hours, and 15.3 % for six to ten hours daily. There were no significant correlations between increased screen time and lack of physical activity or obesity.

### Driving habits

Twenty five per cent of physicians report not using a seatbelt when driving. Speeding was common, with 40.0 % reporting “usually driving beyond the speed limit on the highway (>100 km/h)”. Speeding was more common among the younger age groups (P = 0.015). Physicians who use the phone while driving comprised 39 % of the sample, with a higher rate of phone use (46.8 %) reported by female physicians (P = 0.006). Texting and using social media applications while driving was reported by 19.1 % of the sample.

## Discussion

Numerous studies have demonstrated that the health of medical professionals may affect the health of the larger population, and there is an established association between physicians’ own healthy practices and their patient interactions [18]. Organizations are therefore increasingly adopting approaches to improve physician health [19]. The majority of research into physicians’ health deals with stress, burnout, depression, and substance use, and there has been recent demand for research into lifestyle habits of physicians including exercise, nutrition, and smoking [20].

This study is one of the first to assess lifestyle habits and wellbeing among physicians in the Middle East. The results suggest that, especially in this region, more effort should be put into educating and assisting physicians to make healthier lifestyle changes.

The prevalence of self-reported hypertension was comparable among both physicians (20.3 %) and the general population (age group 30–65: 19.6 %). In the same age group however, the rate of self-reported diabetes mellitus was considerably lower (11.0 % vs 14.5 %). When hypertension and diabetes were measured objectively among the general Bahraini population, the actual rates were found to be 18.1 % (diabetics) and 43.6 % (hypertensives) [3]. A comparison of lifestyle habits and wellbeing between physicians and the general population is represented in Table 3. Physicians are known to be neglectful of their own health [21]. One Irish study found that 30 % of general practitioners had not been to a doctor in the previous five years [22]. In our study, even with the high mean population age, only 3 out of 152 physicians had seen a doctor in the previous six months to check for chronic disease.

**Table 3:** Comparison of Wellbeing and lifestyle habits between physicians and the General population

		General Population 2010^3^ Age group 30-65	Physicians (current study)
		(%)	(%)
Age Groups	30-39	35.3	36.6
	40-49	39.0	29.0
	50-59	21.1	24.1
	60-65	4.5 %	10.3
Gender^a^	Male	48.8	32.9
	Female	51.2	67.1
Heart Disease (self reported)	Yes	-	3.4
	No		96.6
Diabetes Mellitus (self reported)	Yes	14.5	11.0
	No	85.5	89.0
Measured Diabetes Mellitus	Yes	18.1	-
	No	81.9	
Hyperlipidaemia (self reported)	Yes	-	25.5
	No		74.5
Measured Hypercholestrolaemia (>5.2 mmol/l)	Yes	44.9	-
	No	55.1	
Hypertension (self reported)	Yes	19.6	20.3
	No	80.4	79.7
Measured Hypertension (>140/90)	Yes	43.6	-
	No	56.4	
Hereditary Blood Disease (self reported)	Yes	-	15.8
	No		84/2
Physical activity		>10 min(m)/day	>30 min/week
	Yes	40.0	29.6
	No	60.0	70.4
Cigarette Smoking	Never Smoker	79.9	93.2^18^
	Previous Smoker	7.9	4.1
	Current Smoker	12.1	2.7^17^
Waterpipe Smoking	Never Smoker	92.8	93.8
	Current Smoker	7.2	6.2
Total Tobacco Smokers		18.5	8.6^18^
Alcohol Consumption [28]	Lifetime Abstainers	85.6	98.0
	Previous Drinkers	4.6	1.3
	Current Drinkers	9.8	0.7
Mean Sleeping Hours		-	5.9 (±1.2)
BMI Categories	Normal or Low (<24.9)	23.9	27.5
	Overweight 25–29.9)	34.8	39.4
	Obese (>30)	41.3	33.1
Mean BMI (±SD)^a^		28.5 (±6.3)	27.8 (±4.6)

^a^Data provided for age groups 20–65

According to the most recent WHO statistics, Bahrain ranks 15th worldwide in terms of body mass index, with 61.2 % of its adult population having an above normal BMI [23]. In our study, 72.5 % of physicians had an above normal BMI, with significantly higher BMI in older and male physicians. These figures are alarming. They are among the highest reported figures in physicians worldwide; significantly higher than for physicians in other countries including the United States [24], where obesity is considered a substantial public health challenge.

The CDC recommends a minimum of 150 min of moderate to vigorous physical activity per week [25]. Only around 4 % of physicians in our study meet this criterion for leisure time physical activity. It is difficult to compare physical activity between physicians and the general population due to differences in the tools used. Nonetheless, the rate of exercise in Bahraini physicians is extremely low, with only 29.6 % of reporting at least 30 min of exercise during a week. Among the general population (age groups 30–65), 40.0 % reported at least 10 min of exercise during a usual day [2].

Sleep deprivation has been shown to affect physician clinical performance and cognitive scores [26]. Although primary care physicians in Bahrain enjoy a more relaxed timeframe than most other specialisations, the average reported sleeping time for physicians in our study is 5.9 h (SD = 1.2). Physicians who sleep more than 6 h daily were less likely to be smokers and more likely to consume breakfast on most days of the week. This association may demonstrate the clustering of multiple “healthy” lifestyle habits among certain physicians.

Poor lifestyle habits among both physicians and the general population in Bahrain and other neighbouring countries reflect entrenched cultural patterns present in the Middle Eastern population that may be exaggerated among physicians due to excessive workload and long shifts. Consequently, public health services and policy makers in Bahrain have focused interventions on managing NCD complications rather than risk factors. We believe physician health aid programs [27] and even simple institutional interventions have the potential to make a substantial difference in the lifestyle habits of physicians in our region.

Limitations include the timing of the study. We administered the questionnaires during the summer, when extreme temperatures might lead to lower rates of physical activity. Physicians also might have reported inaccurate data because of poor recall or social acceptability bias, potentially causing an underestimation of unfavourable lifestyle habits among physicians assessed in this study. In addition, we used a locally-developed questionnaire, which makes it difficult to accurately compare our data with other studies worldwide. Since we have identified relevant basic lifestyle patterns, we suggest that future studies in Bahrain use standardised questionnaires to comprehensively measure wellbeing, diet, sleep, stress, and physical activity.

## Conclusion

We have demonstrated significantly elevated rates of obesity, low physical activity, and other unfavourable lifestyle habits among physicians. Poor physician wellbeing in Bahrain could have a negative impact on physician-patient interaction. We encourage institutions to be more proactive in improving the health of physicians and healthcare professionals.

## Additional file

Additional file 1:
**Frequency of consumption of different food items.**

## Abbreviations

CDC: Centers for disease control and prevention
NCD: Non-communicable disease
