# Supplementary material for: The lifestyle habits and wellbeing of physicians in Bahrain: a cross-sectional study
Source: BMC Public Health. 2015 Jul 14;15:655. doi: 10.1186/s12889-015-1969-x (PMC4499902; doi:10.1186/s12889-015-1969-x)
Supplement: Additional file 1: — Frequency of consumption of different food items. [file 12889_2015_1969_MOESM1_ESM.docx]

Supplementary material

Frequency of consumption of different food items

| **Food Item** | **Frequency of Consumption* (days/week)** | | | |
| --- | --- | --- | --- | --- |
|  | **Doesn't consume it weekly** | **1-2 days per week** | **3-5 days/week** | **6-7 days/week** |
| Chicken | N=2 (1.4%) | N=61 (41.2%) | N=76 (51.4%) | N=9 (6.9%) |
| Meat | N= 34 (23.0%) | N= 82 (55.4%) | N=30 (23.3%) | N=2 (1.4%) |
| Fish | N=19 (12.8%) | N=90 (60.4%) | N= 36 (24.2%) | N= 4 (2.6%) |
| Fruits | N=4 (2.8%) | N=17 (11.7%) | N=51 (53.2%) | N=73 (50.3%) |
| Vegetables | N=1 (0.7%) | N=8 (5.4%) | N=42 (28.6%) | N=147 (56.3%) |
| * Exact Consumption amount was not specified in the question. | | | | |
